# Supplementary material for: The Wolbachia WalE1 effector alters Drosophila endocytosis
Source: PLoS Pathog. 2024 Mar 28;20(3):e1011245. doi: 10.1371/journal.ppat.1011245 (PMC11003677; doi:10.1371/journal.ppat.1011245)
Supplement: S1 Fig — WalE1 with a 6xHis tag, expressed and purified from E. coli shown as positive control in lane 5. Wolbachia bacteria were isolated from JW18 cells using cell disruption and differential centrifugation (wMel-iso) [24] or the full lysate was used for the western blot (lysate). (PDF) [file ppat.1011245.s001.pdf]

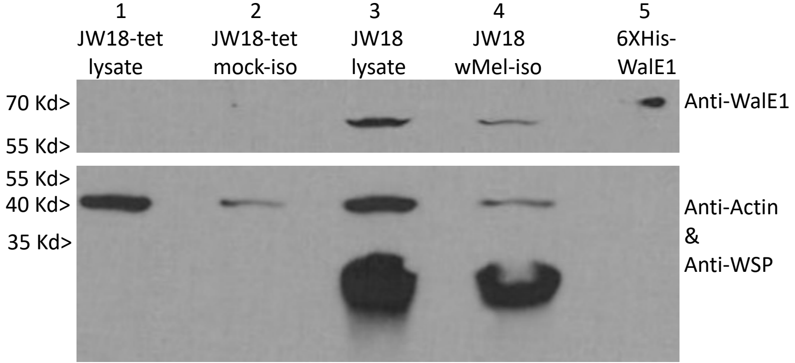
1
JW18-tet
lysate
2
JW18-tet
mock-iso
3
JW18
lysate
4
JW18
wMel-iso
5
6XHis-
WalE1
70 Kd>
55 Kd>
Anti-WalE1
55 Kd>
40 Kd>
35 Kd>
Anti-Actin
&
Anti-WSP
