# Supplementary figures and images for: The Wolbachia WalE1 effector alters Drosophila endocytosis
Source: PLoS Pathog. 2024 Mar 28;20(3):e1011245. doi: 10.1371/journal.ppat.1011245 (PMC11003677; doi:10.1371/journal.ppat.1011245)

DB only

Past1

AD only

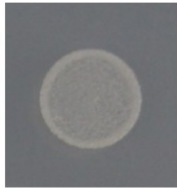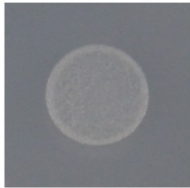

WalE1

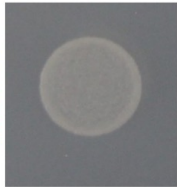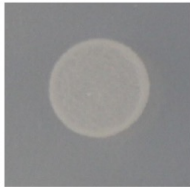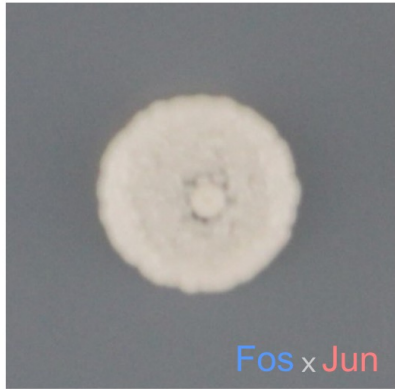

Fos x Jun

Supplement: S2 Fig — Constructs for Past1 and WalE1 were co-expressed in diploid yeast to drive expression of a metabolic marker (HIS3) that allows growth on media lacking histidine. Although our positive control (Fos x Jun) grew, hybrid yeasts with WalE1 and Past1 grew similarly to vector alone (AD only or DB only). (PDF) [file ppat.1011245.s002.pdf]

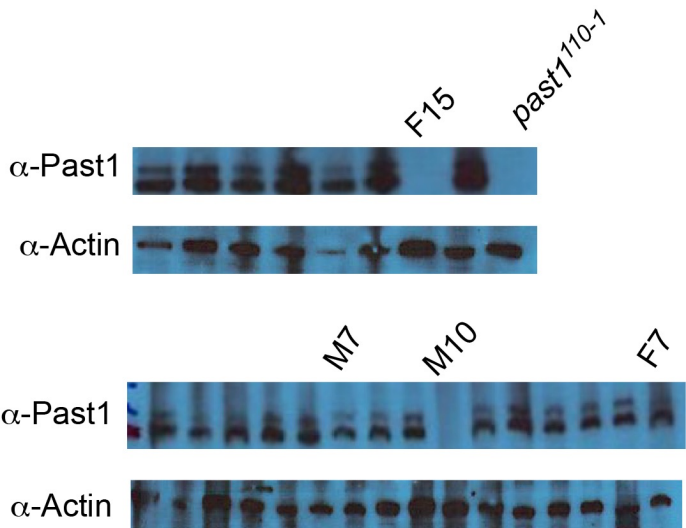

Supplement: S3 Fig — (PDF) [file ppat.1011245.s003.pdf]

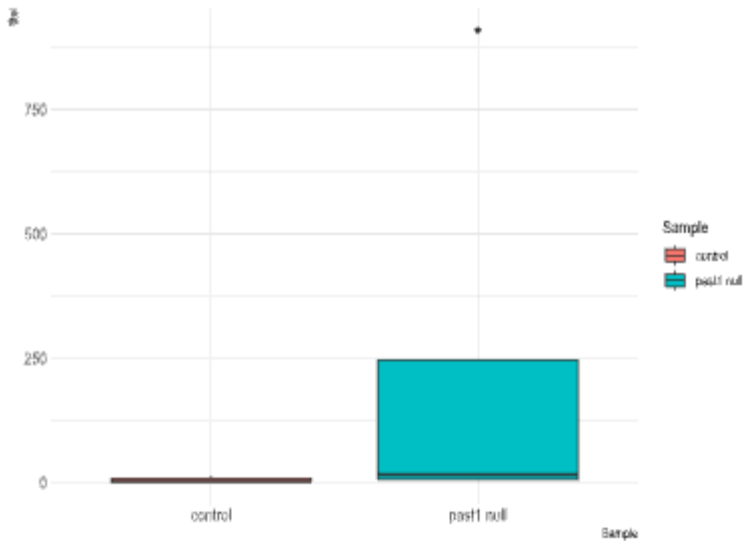

Supplement: S4 Fig — Wolbachia load was measured using qPCR targeting the wsp locus (relative to host gene rpl32). Past1 null flies have a larger relative abundance of Wolbachia compared to wild type flies. (PDF) [file ppat.1011245.s004.pdf]
